# A Remarkable Case

**Published:** 1877-09

**Authors:** G. R. Thomas


### A REMARKABLE CASE.

BY G. R. THOMAS, D. D. S.

In the February number of the REGISTER of this year, an article appears by J. Clark Scott, D. D. S., entitled, "A Strange Case," and asked, "Is there a parallel?"

At the close of his article he refers to a case in Detroit which came under my observation, and was of so much interest that I feel it my duty to report it, especially since Dr. Clark asks for a parallel.

On the 20th of April, 1876, Mrs. T———, of Detroit, called to consult me in relation to a trouble she was then having with her jaw, which, upon examination, presented a case of incomplete anchylosis of the inferior maxilla. The history of the case thus far, in brief, was that about five weeks previous, a dentist of this city was called upon to extract the right inferior *dens sapientia*. The patient was a lady about twenty years of age, with all her teeth in position. This tooth was already erupted, but for lack of room was causing so much disturbance that it was thought best to remove it. Extraction was attempted by pushing an elevator between it and the second molar, using the latter for a fulcrum. The effect was supposed to be successful, but upon searching for the missing tooth it could not be found. She was induced to a partial belief that she had swallowed it. One thing was certain, it had disappeared from its socket. The muscles began to contract at once; the jaw gradually became set, and much pain and suffering was the result for five weeks, during which time she was receiving treatment from her physician.

After giving the case a careful diagnosis, she was dismissed, without prescribing, with instrution to come again the next day, during which time I called upon the dentist who had been employed, who gave substantially the same statement of the case as the patient had. I said to him, such being the facts, I should refer her back to him when she should come to my office next day. He said, "No. If you can cure her, go on and do it." He said she had swallowed the tooth, he had no doubt of it. I commenced the treatment with that view of the case. All the nourishment she could receive was in a liquid form, she not being able to open her teeth more than one eighth of an inch. I immediately commenced the treatment, using gentle force to open the jaws, and gave her a small wooden wedge with notches cut in its surface for her to use at intervals, at the same time using an ointment containing potassæ nitras for external use, and put her upon potassæ bromide. This treatment was continued for about six weeks, with only partial success as a result, she being able to open her mouth without help only about one inch. All this time there kept up a light watery fluid discharge from the socket of the missing tooth. The second molar became loose, and I extracted it. Becoming convinced of the presence of necrosed bone at or near the angle of the jaw, I called in Dr. Thomas A. McGraw, one of our best surgeons, and his diagnosis co-incided with my own.

It was then decided that an operation would be necessary, and she was placed under an anæsthetic, the mucous membrane thoroughly cut away, and the outer portion of the alveolar process was found badly necrosed and cut away. In passing in the fingers to make certain that all the necrosed bone had been cut away, I discovered, the length of the index finger distant, a little in and upward, what appeared to be a loose piece of bone. And this was the first time that I had any idea of the presence of that missing tooth. We were not long in deciding that it must come away, whatever it was. We had no instruments that would reach it, and, strange as it may appear, I did the very thing, without any knowledge of its ever having been done before, that Dr. Scott reports having done the year before, stepped into my laboratory, turned up a rude hooked shaped instrument and was soon rewarded by having the "swallowed *dens sapientiæ*" in my possession, that I now fished out with the said instrument.

It had doubtless been driven by the elevator up and back underneath the mucous membrane, and finally found lodgment between the internal pterygoid muscle and the jaw, where it was finally anchored. The patient commenced to recover at once, and in three months from the date of the operation was considered well, although there was at that time some considerable tenderness about the parts involved.

This is the second case that I know of, on record or otherwise, and I hope never to know of another. No person can get any idea of the real suffering induced by such a case, without having seen it in its various stages. I understand a law suit was the result of the case reported by Dr. Scott, the result of which I have not learned.

